# The Role of the Endogenous Opioid System in the Vocal Behavior of Songbirds and Its Possible Role in Vocal Learning

**DOI:** 10.3389/fphys.2022.823152

**Published:** 2022-02-22

**Authors:** Utkarsha A. Singh, Soumya Iyengar

**Affiliations:** National Brain Research Centre, Manesar, India

**Keywords:** songbirds, endogenous opioids, opioid receptors, learning, reward, basal ganglia

## Abstract

The opioid system in the brain is responsible for processing affective states such as pain, pleasure, and reward. It consists of three main receptors, mu- (μ-ORs), delta- (δ-ORs), and kappa- (κ-ORs), and their ligands – the endogenous opioid peptides. Despite their involvement in the reward pathway, and a signaling mechanism operating in synergy with the dopaminergic system, fewer reports focus on the role of these receptors in higher cognitive processes. Whereas research on opioids is predominated by studies on their addictive properties and role in pain pathways, recent studies suggest that these receptors may be involved in learning. Rodents deficient in δ-ORs were poor at recognizing the location of novel objects in their surroundings. Furthermore, in chicken, learning to avoid beads coated with a bitter chemical from those without the coating was modulated by δ-ORs. Similarly, μ-ORs facilitate long term potentiation in hippocampal CA3 neurons in mammals, thereby having a positive impact on spatial learning. Whereas these studies have explored the role of opioid receptors on learning using reward/punishment-based paradigms, the role of these receptors in natural learning processes, such as vocal learning, are yet unexplored. In this review, we explore studies that have established the expression pattern of these receptors in different brain regions of birds, with an emphasis on songbirds which are model systems for vocal learning. We also review the role of opioid receptors in modulating the cognitive processes associated with vocalizations in birds. Finally, we discuss the role of these receptors in regulating the motivation to vocalize, and a possible role in modulating vocal learning.

## Introduction

**FIGURE 1:**
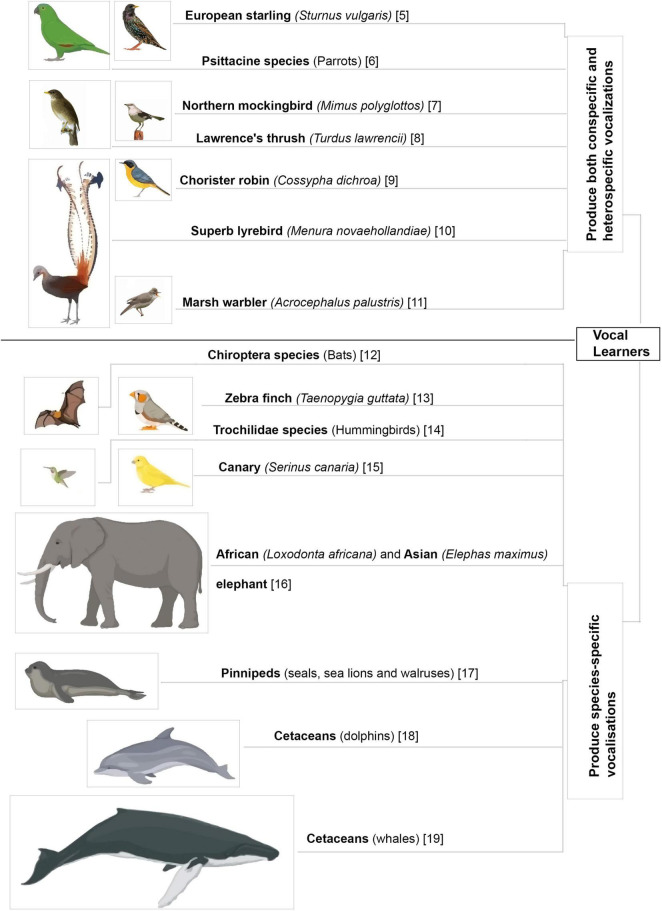
Different animal species that learn to vocalize. Vocal mimics further enhance their vocal repertoires by including heterospecific and environmental sounds in their vocalizations (Harcus, 1977; Eens, 1997; Hardy and Parker, 1997; Dowsett-Lemaire, 2008; Gammon and Altizer, 2011; Balsby et al., 2012; Janik, 2014; Mello, 2014; Reichmuth and Casey, 2014; Stoeger and Manger, 2014; Favaro et al., 2016; Mori et al., 2018; Johnson and Clark, 2020; Vernes and Wilkinson, 2020; Dalziell et al., 2021).

Acoustic communication is important for the survival of animals living in large social groups. Many species of animals utilize innate vocalizations to relay information to others. The context for such innate vocalizations may be acquired from the environment (DeVries et al., 2015; Wegdell et al., 2019) but is not affected by changes in auditory input during early development (Cheney et al., 1992; Hammerschmidt et al., 2012). In contrast, some animals have elaborate vocal repertoires which are learnt during the course of development and even in adulthood (Figure 1; Harcus, 1977; Eens, 1997; Hardy and Parker, 1997; Dowsett-Lemaire, 2008; Gammon and Altizer, 2011; Balsby et al., 2012; Janik, 2014; Mello, 2014; Reichmuth and Casey, 2014; Stoeger and Manger, 2014; Favaro et al., 2016; Mori et al., 2018; Johnson and Clark, 2020; Vernes and Wilkinson, 2020; Dalziell et al., 2021). Neuroethologists study these vocal learners to understand the intricacies and origins of human speech acquisition.

One such avian species which learns to produce complex vocal signals are songbirds. Like humans, these birds live in intricate social groups wherein vocal communication is essential (Zann, 1996; Fernandez et al., 2017; Tchernichovski et al., 2017). Birdsong is used to attract mates, mark territories and is the defining feature of songbirds, refined across generations by sexual selection (Zann, 1996). One of the first studies that hinted at song learning in passerine birds was performed by Barrington (1773). In a letter to the Royal Society of London, he wrote about the stages of song learning in young birds, differentiating begging calls for food, distance calls for communication and learnt vocalizations. He stated that the initial phase of song production in young birds was very similar to babbling in human babies, which was strongly influenced by the social environment (Barrington, 1773). Furthermore, active tutors were found to be essential for both young songbirds and human babies. Earlier studies had demonstrated that human babies did not learn language from audio/video recordings (Kuhl et al., 2003). Similarly, Baptista and Petrinovich (1984, 1986) showed that white crowned sparrows (*Zonotrichia leucophrys*) tutored with taped songs of conspecific adult males until 50 days post hatch chose to learn the songs of a live heterospecific tutor (strawberry finches, *Amandava amandava*) presented to them after the sensitive period for learning ended. These findings suggested that vocal learners chose to learn from a live heterospecific tutor rather than from the taped vocalizations of conspecifics, showing that social interactions were important for vocal learning.

The presence of a strong social influence and an internal reward guiding vocal learning [reviewed in Riters (2011) and Riters et al. (2019)] hints at the involvement of neuromodulators in vocal learning during the sensitive period. One of the most potent neuromodulator groups that are associated with social reward and motivation is the endogenous opioid system. It is composed of opioid receptors (ORs) and their ligands, the endogenous opioid peptides. The four primary subtypes of opioid receptors include μ (mu)-ORs encoded by the OPRM1 gene (Chen et al., 1993a; Fukuda et al., 1993; Wang et al., 1993), δ (delta)-ORs encoded by the OPRD1 gene (Evans et al., 1992; Kieffer et al., 1992), κ (kappa)-ORs encoded by OPRK1 gene (Chen et al., 1993b; Meng et al., 1993; Minami et al., 1993; Yasuda et al., 1993) and Nociceptin/Orphanin FQ receptors encoded by the OPRL1 gene (Fukuda et al., 1994; Mollereau et al., 1994; Wang et al., 1994; Table 1). The present review focuses mainly on the two most studied subtypes, that is, the μ- and δ-ORs. Besides being activated by their respective endogenous ligands (Pleuvry, 1991), endorphins and enkephalins, respectively, the ORs also bind to a lesser degree to the other opioid ligands (Jordan et al., 2000). The endorphins are synthesized after post-translational modification of the precursor prohormone preproopiomelanocortin (POMC; Smyth, 1983), whereas δ-ORs are activated by enkephalins derived from the peptide precursor preproenkephalin (PENK; Hughes et al., 1975). A third type of opioid ligand, the dynorphins, synthesized from prodynorphin bind to κ-ORs (Goldstein et al., 1979, 1981). By binding to these receptors, opioids influence a variety of physiological activities including analgesia, hunger, motivation, anxiety and even learning (Bodnar, 2004; Wilson and Junor, 2008; Kibaly et al., 2019). Whereas research on opioid addiction has largely overshadowed the role of these receptors in higher cognition, recent studies have shown their involvement in learning (Meilandt et al., 2004; Bertran-Gonzalez et al., 2013).

**TABLE 1:** Opioid peptide receptors and their ligands.

Name*	Endogenous ligand(s)	Action	pKi
**μ, mu or MOP**	β-Endorphin	Full agonist	9 (Raynor et al., 1994)
	Leu-Enkephalin	Partial agonist	8.1 (Toll et al., 1998)
	Met-Enkephalin	Full agonist	9.2 (Raynor et al., 1994)
	Endomorphin-1	Potential full agonist	8.3 (Zadina et al., 1997; Gong et al., 1998)
	Endomorphin-2	Potential full agonist	8.5 (Zadina et al., 1997)
	Dynorphin A	Full agonist	8.3 (Toll et al., 1998)
	Dynorphin B	Full agonist	8.5 (Toll et al., 1998)
**δ, delta or DOP**	Leu-Enkephalin	Full agonist	8.4 (Raynor et al., 1994)–8.7 (Toll et al., 1998)
	β-Endorphin	Full agonist	8.3 (Toll et al., 1998)–9 (Raynor et al., 1994)
	Met-Enkephalin	Full agonist	6.0 (Meng et al., 1993)
	Dynorphin A	Full agonist	7.8 (Toll et al., 1998)
	Dynorphin B	Full agonist	7.8 (Toll et al., 1998)
	Endomorphin-1	Potential full agonist	6.1 (Zadina et al., 1997)
**κ, kappa or KOP**	Dynorphin A	Full agonist	8.3–10.8 (Simonin et al., 1995; Zhu et al., 1995, 1997; Toll et al., 1998)
	Dynorphin B	Full agonist	8.1–9.9 (Meng et al., 1993; Simonin et al., 1995; Toll et al., 1998)
	Leu-Enkephalin	Full agonist	6.8 (Meng et al., 1993)
	Met-Enkephalin	Partial agonist	6.3 (Simonin et al., 1995)–7.9 (Toll et al., 1998)
	α-Neoendorphin	Full agonist	8.3–10.2 (Li et al., 1993; Meng et al., 1993; Simonin et al., 1995; Zhu et al., 1995)
**NOP**	Nociceptin/orphanin FQ	Full agonist	8.4–10.4 (Adapa and Toll, 1997; Dooley et al., 1997; Bigoni et al., 2002)

*NC-IUPHAR (Nomenclature and Standards Committee of the International Union of Basic and Clinical Pharmacology) -approved nomenclature. DOP, delta opioid receptor; KOP, kappa opioid receptor; MOP, mu opioid receptor; NOP, nociceptin opioid receptor.

Both μ- and δ-ORs can act to modulate different kinds of learning, with some researchers hypothesizing that these receptors help in learning the association between drug and reward in addiction (Klenowski et al., 2015). In the present review, we will discuss the anatomical distribution of opioid ligands and receptors in the brain of songbirds and how these receptors may modulate vocalization and vocal learning.

## Vocal Learning and the Underlying Neural Circuitry

The process of vocal learning begins with the perception of adult vocalizations. Human babies are exposed to language *in utero* and newborns respond more to their mother’s voice and language (DeCasper and Fifer, 1980; Moon et al., 1993). This suggests that before learning semantics and grammar, babies learn to identify phonetic arrangements specific to their native language. Songbirds too begin to learn parental vocal signals early in development. The superb fairy wren (*Malurus splendens*) learns its mother’s incubation calls *in ovo* and uses a similar vocal structure in its own begging calls for food (Colombelli-Negrel et al., 2014). This has been tested by showing that the embryos of this species of birds show an increased heart rate in response to a playback of tutor songs *in ovo* (Colombelli-Negrel and Kleindorfer, 2017).

After hatching, the young birds begin an early phase of learning is called the *sensory phase*, during which they memorize their tutor’s songs. An increased response to the playback of a known song in white-crowned sparrows, trained using songs taped from the tutor, suggests the presence of a memory of the imitated song (Nelson, 1997). Once the song template is learnt, young birds begin to sing a soft and immature subsong (Immelmann, 1969). This is the sensorimotor phase during which the bird tries to match its own song to the “mental template” it had acquired during the sensory phase. Auditory feedback helps in matching the bird’s own vocalizations to that of their fathers/tutors (Konishi, 1965; Brainard and Doupe, 2000). With practice, the vocalizations of the young bird become more structured, but still possess the ability to undergo change. These vocalizations are called plastic songs, which finally develop into a fully structured unchangeable vocal pattern in adulthood (Eales, 1985; Slater and Jones, 1998). For closed-ended learners such as zebra finches (*Taenopygia guttata*) and white crowned sparrows (*Zonotrichia leucophrys*), the adult song does not undergo further change and is aptly referred to as “crystallized song” (Immelmann, 1969; Marler and Peters, 1982; Böhner, 1990; Zann, 1990).

**FIGURE 2:**
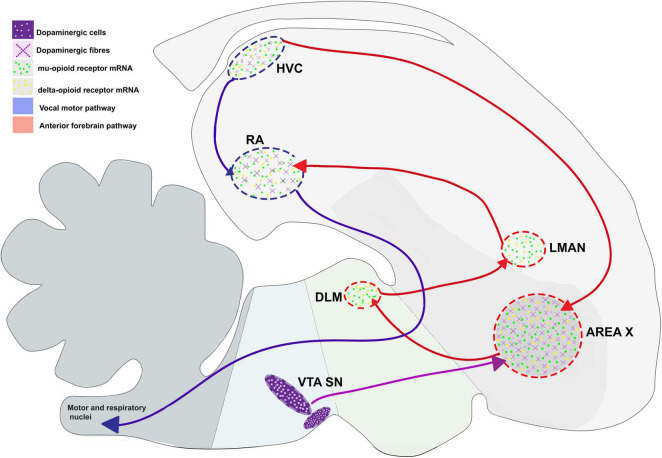
Expression pattern of μ- and δ-opioid receptors and the dopaminergic system in the vocal control nuclei in the songbird brain, based on data from Bottjer (1993) and Khurshid et al. (2009). Blue arrows represent connections of HVC connections, red arrows represent connections of AFP, and violet arrows represent dopaminergic input. DLM, dorsolateral nucleus of the medial thalamus; LMAN, lateral magnocellular nucleus of the anterior nidopallium; RA, robust nucleus of the arcopallium; SN, substantia nigra; VTA, ventral tegmental area.

For songbirds, the process of vocal learning and production is controlled by specific brain areas called song control nuclei. Nottebohm et al. (1982) showed the presence of five such nuclei in the songbird brain that were associated with vocal control. These nuclei included the pallial sensorimotor nucleus HVC (used as a proper name) which projects to a pallial motor nucleus RA (robust nucleus of the arcopallium) in the caudal part of the brain and forms the vocal motor pathway (VMP), which is important for vocalization since it controls the movements of the syrinx (Nottebohm and Arnold, 1976; Nottebohm et al., 1982). A nucleus in the avian basal ganglia, Area X, also receives projections from HVC and projects to the thalamic nucleus DLM (dorsolateral nucleus of the medial thalamus), which in turn projects to LMAN (lateral magnocellular nucleus of the anterior nidopallium, LMAN). The pathway connecting Area X, DLM and LMAN forms a thalamocortical basal ganglia loop called the anterior forebrain pathway (AFP; Nottebohm et al., 1976; Bottjer et al., 1989), responsible for vocal learning (Bottjer et al., 1984; Scharff and Nottebohm, 1991). The two pathways are interconnected via projections from LMAN to RA (Figure 2; Herrmann and Arnold, 1991).

## Signaling and Neural Expression of the Endogenous Opioid System

The opioid receptors are members of the G-protein coupled receptor (GPCR) family. They are composed of seven hydrophobic transmembrane domains connected via intra and extra-cellular loops and possess N- or amino and C or carboxylic groups at the end terminals. Structurally, the μ- and δ-ORs have a binding pocket that interacts with the respective ligand and specifically recognizes the morphinan group (Table 1; Mansour et al., 1997; Granier et al., 2012). Following activation, μ-ORs inhibit adenyl cyclase and voltage-gated Ca^2+^ channels, and stimulate G protein-activated inwardly rectifying K+ channels (GIRKs) and phospholipase Cβ by the activation of the G_αi/o_ and G_βγ_ subunits (Childers, 1991). Similarly, for δ-ORs, the activation of the G_αi/o_ and G_βγ_ subunits results in modulation of the activity of calcium channels (P/Q-, N-, and L-type), GIRKs, and inhibition of adenylyl cyclase which reduces the level of intracellular calcium via inhibition of cAMP-dependent calcium channels. Together, these events result in the inhibition of neural activity (Kieffer and Evans, 2009; Al-Hasani and Bruchas, 2011; Gendron et al., 2016). Once activated, both μ- and δ-ORs are internalized and δ-ORs are specifically degraded via the lysosomal pathway (Whistler et al., 2002). In contrast, μ-ORs may continue to be involved in signal transduction even after internalization and only unbound μ-ORs are recycled (Al-Hasani and Bruchas, 2011).

### μ-ORs and Their Ligands

In mammals, μ-ORs and their ligands (enkephalins and endorphins) are mostly concentrated in the hippocampus, thalamic nuclei, amygdala, locus coeruleus, parabrachial nucleus, and the nucleus of the solitary tract (Bloom et al., 1978; Di Giulio et al., 1979; Gall et al., 1981). Within the striatum, these receptors are concentrated in patches, which are embedded in a matrix intensely stained for acetylcholine and rich in Substance P (Pert et al., 1976; Brimblecombe and Cragg, 2017). Although the patch and matrix organization of the mammalian striatum is absent in birds, levels of μ-ORs are uniformly high across the striatum in birds including pigeons (*Columba livia*) (Reiner et al., 1989), chick (*Gallus gallus*) (Csillag et al., 1990), juncos (*Junco hyemalis*) (Gulledge and DeViche, 1995; Gulledge and Deviche, 1999) and zebra finches. Furthermore, μ-ORs are present across development in some of the song control regions of juncos HVC, RA, LMAN, and Area X (Gulledge and DeViche, 1995; Gulledge and Deviche, 1999), especially in RA, and in all song control nuclei including HVC, RA, LMAN, Area X, and DLM in adult male zebra finches (Figure 2; Khurshid et al., 2009).

Comparatively fewer studies have been conducted to detect opioid ligands in songbirds. Initial studies used specific antibodies to detect the presence of leu-enkephalin (Ryan et al., 1981) and met-enkephalin (Ryan et al., 1981; Bottjer and Alexander, 1995) in different song control nuclei. Both reports demonstrated the presence of enkephalinergic fibers and somata in components of the VMP and AFP in adult male zebra finches. Additionally, Carrillo and Doupe (2004) found that medium spiny neurons as well as large DLM-projecting neurons in Area X were immunoreactive for leu-enkephalin. These findings were confirmed by Xie et al. (2010), who used mass spectrometry and MALDI-TOF to demonstrate that both proopiomelanocortin (POMC), the precursor peptide of β-endorphin, and preproenkephalin (PENK, the precursor of enkephalin) were present in Area X, LMAN, HVC, and RA.

The presence of both POMC and PENK as well as μ-ORs in components of the VMP (HVC→RA) and AFP (the neural circuit connecting LMAN, Area X, and DLM) in songbirds suggests that they may be able to modulate both vocalization and vocal learning and/or singing in different social contexts. In particular, the localization of μ-ORs in the avian striatum suggests that these receptors may be important for the control of reward-guided behavior, since undirected singing and song learning (cf., Scharff and Nottebohm, 1991) during practice is thought to be internally rewarding (Riters et al., 2019).

### δ-ORs and Their Ligands

The neural expression of δ-ORs has been well-documented in rodents. Developmentally, δ-OR expression begins in the pons and the hypothalamus at embryonic day 13 (E 13.5), whereas μ- and κ-ORs are detected in the basal ganglia and midbrain at E 11.5. The prenatal expression of δ-ORs at day E 17.5 and E 19.5 is very low and restricted to the caudate putamen, parabrachial nucleus and olfactory tubercle (Zhu et al., 1998). In contrast, in adult rodents, δ-ORs are distributed in the olfactory tubercle, cerebral cortex, amygdala, nucleus accumbens, and striatum (Mansour et al., 1987, 1994) and have a low, yet detectable presence in the hippocampus and VTA (Erbs et al., 2015). Since this pattern of expression is absent in the prenatal stages, it is possible that they may influence the development of neural circuits developing after birth and modulate the associated cognitive processes. Furthermore, the expression of δ-ORs in areas regulating reward, motivation, learning, memory, and emotional processing (Jutkiewicz, 2018) suggests that these receptors may be involved in cognitive processes such as vocal learning.

In songbirds (zebra finches), the expression of enkephalin is similar to that of δ-ORs. Neuronal fibers immunoreactive for met-enkephalin are distributed across the pallium and are concentrated in song control areas including HVC, RA, LMAN, Area X, and DLM (Ryan et al., 1981; Bottjer and Alexander, 1995). Patterns of δ-OR expression mirrored these results, with δ-OR mRNA being localized to the song control nuclei (LMAN, HVC, RA), all parts of the pallium and hippocampus, and Area X expressing the highest levels of these receptors in adult male zebra finches (Khurshid et al., 2009; Parishar et al., 2021).

As in rodents (see above), it is possible that the expression of δ-ORs and their ligands (enkephalins) may be developmentally regulated in songbirds as well. An earlier study has found higher levels of δ-OR expression in Area X and RA of juvenile juncos (Gulledge and DeViche, 1995), whereas Carrillo and Doupe (2004) have shown that higher levels of leu-enkephalin are present in Area X in juvenile zebra finches, compared to those in adult birds of both species. Another study by Wada et al. (2006) demonstrated that singing for a 30-min duration led to the expression of preproenkephalin (PENK, the precursor of enkephalin) in the song control regions HVC and Area X in adult and juvenile male zebra finches. Furthermore, the expression of PENK (which primarily binds δ-ORs) as well as mRNA for μ-ORs was upregulated in the mPOA (medial preoptic nucleus) in male European starlings (*Sturnus vulgaris*) during fall, a season marked by increased undirected singing (Riters et al., 2014). The mPOA is connected to the ventral tegmental area (VTA) in male starlings, and lesions of this nucleus lead to deficits in the motivation to sing to females and other courtship-associated behaviors (Riters and Ball, 1999; Alger et al., 2009).

Taken together, these findings suggest that the endogenous opioid system may be involved in modulating song-induced reward associated with positive affect [reviewed in Riters (2011) and Riters et al. (2019)] and may also be involved in guiding vocal learning during development.

## The Role of the Endogenous Opioid System in Vocalization and the Motivation to Sing

Besides their involvement in reward and motivation in mammals, μ-ORs are also important for socialization and singing in birds. Female-directed (FD) singing is a highly motivated behavior in which both the mesolimbic dopaminergic systems as well as the opioid system are involved. Recent studies have shown that blocking dopamine receptors using antagonists leads to a decrease in courtship singing in zebra finches (Schroeder and Riters, 2006; Rauceo et al., 2008). The expression of endogenous opioids has been demonstrated in song control nuclei and areas important for motivation and reward in zebra finches (Bottjer and Alexander, 1995; Carrillo and Doupe, 2004). Furthermore, systemic administration of high doses of naloxone (a general opioid receptor agonist with higher affinity for μ- compared to δ-ORs) led to small increases in the number of FD songs in male starlings (Riters et al., 2005), whereas there was a significant decrease in their number following injections of the μ-OR agonist, fentanyl (Schroeder and Riters, 2006). In addition to the endogenous opioids, μ-ORs are expressed in the song control nuclei as well as the VTA-SNc complex in adult male zebra finches (Khurshid et al., 2009, 2010). In contrast to their effects on male starlings, systemic administration of low doses of the opioid antagonist naloxone leads to a decrease in both FD and undirected (UD) singing in adult male zebra finches. Despite different results in the two species, both sets of findings suggest that ORs are involved in the motivation to sing. In naloxone-treated birds, the decrease in the motivation to sing was accompanied by changes in the quality of song: spectral features (including goodness of pitch, frequency, and amplitude modulation) decreased, whereas the duration of songs and intersyllable intervals (temporal features) increased in length, compared to vehicle-treated controls (Khurshid et al., 2010).

Studies on starlings by Kelm-Nelson et al. (2013) and Riters et al. (2014) have demonstrated that mPOA is involved in the motivation to sing. Kelm-Nelson and Riters (2013) have demonstrated that high levels of μ-ORs and enkephalin are present in mPOA in birds which are poor singers (Kelm-Nelson et al., 2013). As mentioned above, Riters et al. (2014) have reported an increase in the expression of PENK and μ-OR mRNA in the mPOA of male starlings during undirected singing in fall. More recently, research on male European starlings suggests that there is a correlation between reward associated with singing behavior and opioid-related gene expression in mPOA. These findings have been confirmed by Stevenson et al. (2020), wherein blocking μ-ORs in mPOA leads to a significant decrease in undirected song and hinders the association between singing and a positive affective state.

Recent studies have shown that blocking ORs with naloxone specifically in components of the AFP, as opposed to systemic injections, led to changes in the motivation to sing as well as those in the acoustic features of FD songs in adult male zebra finches (Kumar et al., 2019, 2020). Infusions of naloxone into LMAN (Kumar et al., 2019) resulted in a significant decrease in the number of FD songs (Kumar et al., 2020). Blocking ORs in both LMAN and Area X led to significant decreases in the length of motifs produced during FD song. Whereas blocking ORs in LMAN led to significant decreases in the amplitude modulation of motifs at a specific dose (100 ng/ml) of naloxone (Kumar et al., 2019), the same manipulation in Area X led to significant decreases in frequency and amplitude modulation and pitch goodness as well as a significant increase in pitch (Kumar et al., 2020). Additionally, blocking ORs in LMAN and Area X led to changes in the spectral quality of individual syllables in directed songs. Furthermore, naloxone infusion into Area X resulted in a local increase in dopamine. These results suggest that altering opioid modulation in LMAN and Area X may lead to changes downstream at the level of the ventral tegmental area (VTA) which sends dopaminergic projections to Area X, among other targets (Gale et al., 2008).

## The Role of Opioid Receptors in Learning

### μ-ORs and Learning

Besides playing a role in modulating the motivation to sing as well as the spectro-temporal features of song, both μ- and δ-ORs may play a role in modulating song learning since they are present in components of the AFP, including Area X, LMAN, and DLM (Khurshid et al., 2009; Parishar et al., 2021). These findings are supported by earlier reports demonstrating that these receptors are involved in different kinds of learning.

### Studies in the Rodent Model System

Aloyo et al. (1993) showed that μ-ORs could modulate associative learning. Using Pavlovian conditioning in rabbits, they conditioned the nictitating membrane reflex to an audio tone using an air puff. Whereas intraventricular administration of saline did not interfere with the learning process, intraventricular injections of D-Ala, Me- Phe-, Gly-ol enkephalin (DAMGO), a μ-OR agonist, impaired conditional learning in experimental animals. This effect was blocked by μ-OR antagonist naloxone (Aloyo et al., 1993). Furthermore, Loh and Galvez (2014) demonstrated that opioid modulation regulated associative learning in a rodent model system. They used a trace paradigm – whisker–trace–eye blink (WTEB) conditioning, wherein an eye blink, elicited using a periorbital electric shock, was conditioned to whisker stimulation. They observed that if naloxone was administered before the conditioning, it was capable of significantly impairing associative learning. However, administration of naloxone after learning did not have any effect on the conditional association in this paradigm (Loh and Galvez, 2014).

To further establish that μ-ORs are involved in motor learning, Lawhorn et al. (2009) used the dermorphin-saporin toxin which specifically targets and destroys neurons in the striosomes which express μ-OR in the basal ganglia. When these mice were tested on motor tasks, they showed specific impairments only on the rotarod test. Striosomes project to substantia nigra pars compacta, a major source of dopaminergic input to the striatum, and ablating μ-OR-positive neurons in this region would lead to a decrease in dopamine release. Since dopaminergic feedback from the midbrain to the striatal and cortical circuits may provide the necessary reinforcement needed to learn and perform on the rotarod, its absence would lead to deficits in motor learning (Lawhorn et al., 2009). In contrast to this study, Cominski et al. (2014) has shown that the loss of μ-ORs leads to an increase in hippocampal neurogenesis which in turn facilitates spatial learning. Furthermore, the μ-OR antagonist naltrexone is known to facilitate spatial learning and memory formation in mice by increasing AMPA receptor phosphorylation and membrane insertion (Kibaly et al., 2016). Yet another study (Laurent et al., 2015) has shown that μ- and δ-ORs play important roles in incentive learning and in value-based and stimulus-based decision-making in mice.

Although these learning paradigms cannot be compared directly to vocal learning in birds, striatal-based learning involves different aspects of social association (Carouso-Peck and Goldstein, 2019), timing (Gobes et al., 2019) and cued dopaminergic input (Gadagkar et al., 2016), each of which are important factors for vocal learning.

### Studies on δ-ORs and Learning in the Chicken Model System

Initial studies on the involvement of δ-ORs in learning were performed on chicks (*Gallus gallus*) by training them on a passive avoidance task. In this experiment, birds were provided with steel beads coated with a bitter tasting chemical called methyl anthranilate (MEA), which they are averse to. A different set of birds, used as controls, were presented with steel beads coated with water. Both experimental and control birds were presented with a single steel bead and their latency to peck and aversive behavior after pecking at the bead was measured to estimate their behavioral response. Experimental birds learned to associate the steel bead with the bitter taste and avoided pecking at the other bead whereas control birds did not avoid the bead. Administration of leu-enkephalin and D-Pen–2, L-Pen–5 enkephalin (DPLPE), a δ-OR selective agonist, into the intermediate medial hyperstriatum ventrale 5 min before training resulted in poor performance on this task. This study also demonstrated that the amnesia caused by δ-OR agonists was reversed by administration of δ-OR antagonists (Patterson et al., 1989). Csillag et al. (1993) also used a passive avoidance task similar to that used by Patterson et al. (1989) to establish the role of these receptors in learning. In their paradigm, 1-day old chicks were trained to peck at a chrome bead coated with methyl anthranilate or water (control). Neural tissue from the trained birds was tested for binding with radio-labeled ligands specific for δ-, μ-, and κ-ORs. Interestingly, there was higher binding for the δ-OR ligand 3H-DPDPE in the striatal areas medial striatum (MSt) and lateral striatum (LSt) (Csillag et al., 1993) in birds which performed well on the avoidance task. In another study, site-specific injections of δ-OR antagonist ICI-174,864 in MSt abolished the avoidance learning for the bitter-tasting bead in 1-day old chicks (Freeman and Young, 2000).

### Studies on δ-ORs and Learning in the Rodent Model System

Recent studies on δ-ORs and learning have provided further evidence that these receptors show changes in expression patterns based on the learning experience. Bertran-Gonzalez et al. (2013) trained mice on a Pavlovian instrumental transfer protocol (PIT) in which associations learnt following the delivery of a reward influences the behavior toward two external cues. In this study, a sound was linked to a food reward, followed by training on a second task where a lever press delivered the food reward. A successful test session of the instrumental transfer comprised of the mouse pressing the lever when the sound stimulus was presented. The researchers observed an increase in the expression of δ-ORs in cholinergic interneurons within the shell of nucleus accumbens in mice that had learnt the reward association as well as the instrumental transfer (Bertran-Gonzalez et al., 2013). Interestingly, the level of learning determined the extent of δ-OR expression. These results were similar to those shown in the earlier study by Csillag et al. (1993), which demonstrated an increased binding for δ-ORs in the striatum of chicks trained on an avoidance task.

Another study explored the function of δ-ORs in the hippocampus and their role in learning by using δ-OR gene knockout mice (*Oprd1^–/–^*). These gene-deficient mice were poor in place recognition (hippocampal-based learning). Interestingly, they performed better on tasks involving the striatum, such as balancing on an accelerating rotarod, compared to wild type mice (controls). Peripheral injections of the δ-OR antagonist naltrindole in normal mice were able to produce learning deficits similar to the *Oprd1^–/–^* mice. This study concluded that δ-ORs are involved in hippocampal-based learning and possibly modulate parvalbumin interneurons that regulate long term potentiation (Le Merrer et al., 2013). Recently, Leroy et al. (2017) has demonstrated a more specific role for hippocampal δ-ORs. They injected naltrindole into the CA2 region of the hippocampus of young mice interacting with their mates. The study revealed that blocking δ-ORs impairs social memory formation by a failure to induce plasticity in the CA2 region (Leroy et al., 2017).

Despite these findings, learning and memory can never be separated from internally reinforcing reward signals, making it difficult to isolate the role of opioid receptors in learning and memory formation from reward related processes in the brain. It is therefore possible that opioid receptors might modulate learning and memory not just via inhibition of local circuits but also by regulating dopaminergic signaling.

## Modulation of Dopaminergic Signaling Via μ-ORs: Effects of the Opioid System on the Reward Pathway

The interaction between μ-ORs and dopaminergic signaling has been extensively studied in association with addiction and pain pathways (Van Vliet et al., 1990; Li et al., 2016; Burns et al., 2019). Recently, Galaj et al. (2020) have shown that μ-ORs are highly expressed by the GABAergic neurons of the substantia nigra pars reticulata (SNr), that sends dense innervations to the dopamine rich substantia nigra pars compacta (SNc) and ventral tegmental area (VTA) in mice. They observed that optogenetic activation of SNr GABAergic neurons increased heroin intake and reduced heroin-primed drug seeking, whereas inhibition of these neurons induced optical cranial self-activation and place-preference. These results hint at the opioid modulation of reward via inhibitory projections of the SNr to SNc and VTA (Galaj et al., 2020).

Earlier research shows that δ-OR signaling also influences dopaminergic circuitry and vice versa. Dopaminergic afferents acting via D2 receptors are known to inhibit the production of enkephalin which preferentially binds δ-ORs in striatal neurons (Normand et al., 1988; Jiang et al., 1990; Llorens-Cortes et al., 1991). Conversely, blocking D2 receptors increases the production of enkephalin in the striatum (Steiner and Gerfen, 1999). This coupling between δ-OR and dopaminergic signaling may be responsible for memory retrieval and increased retention of information in mice with induced amnesia (Dubrovina and Ilyutchenok, 1996). Dopaminergic release in the striatum also regulates predictive learning by signaling for error in performance (Iordanova, 2009).

Interestingly, the pharmacological activation of μ-ORs in the ventral striatum induces the activation of δ-OR subtypes, which further enhances dopamine release within the area (Hirose et al., 2005). Opioids can exert control over dopaminergic signaling via different mechanisms. The ORs expressed by GABAergic neurons in VTA or on medium spiny neurons in the striatum lead to the disinhibition of dopaminergic neurons thereby causing an increased release of dopamine. Furthermore, ORs present on dopaminergic neurons in the VTA can inhibit the release of dopamine in the striatum [reviewed in Xi and Stein (2002)]. Taken together, a fine balance between dopaminergic and opioid signaling is required for behavioral reinforcement.

The error prediction and behavioral reinforcement through dopaminergic signaling from the midbrain is essential for vocal learning as well. Evaluative signals in the form of bursts of dopamine released in Area X in zebra finches shape the spectral features of song according to the desired template (Gadagkar et al., 2016; Xiao et al., 2018). Since μ- and δ-ORs are present in components of the AFP (such as LMAN and Area X) which are linked to the VTA-SN complex in birds (Ding and Perkel, 2002; Kumar et al., 2019, 2020), it is possible that the complex interplay between OR activation and dopamine release plays a role in the structuring of vocal patterns during song learning, as discussed below.

## Opioid Regulation of Socially Rewarding Behavior and Vocalizations May Also Be Involved in the Regulation of Vocal Learning

Vocal learning is a social process (Tchernichovski et al., 2017) which is rewarding in itself (Riters and Stevenson, 2012). Whereas rodents do not display socially guided vocal learning, certain vocalizations are associated with socially rewarding behavior (Humphreys and Einon, 1981). One such behavior is social play, wherein rodents emit short bursts of high frequency vocalizations (<0.5 s; ∼ 50 Hz) while playing. A play-associated place preference can be established in rodents (Normansell and Panksepp, 1990), suggesting that it is intrinsically rewarding. Similarly, singing-induced place preference can be established in European starlings and zebra finches singing undirected songs (Riters and Stevenson, 2012), also indicating that undirected song, which is produced during learning and for song maintenance, is intrinsically rewarding [reviewed in Riters et al. (2019)]. This play-associated vocal behavior is also demonstrated when young rodents are placed in a spatial location associated with play behavior (Knutson et al., 1998). Opioid agonists such as morphine increase these play-associated vocalizations when administered chronically (Hamed and Boguszewski, 2018). Furthermore, response to play vocalizations is enhanced by opioid agonists and reduced by antagonists (Wohr and Schwarting, 2009). These findings suggest that opioids enable behaviors associated with social activity. In case of a social learning process, like vocal learning, this could help in shaping vocal structure by directing attention toward adult vocalizations produced by tutors and/or other members of the flock (Chen et al., 2016).

**FIGURE 3:**
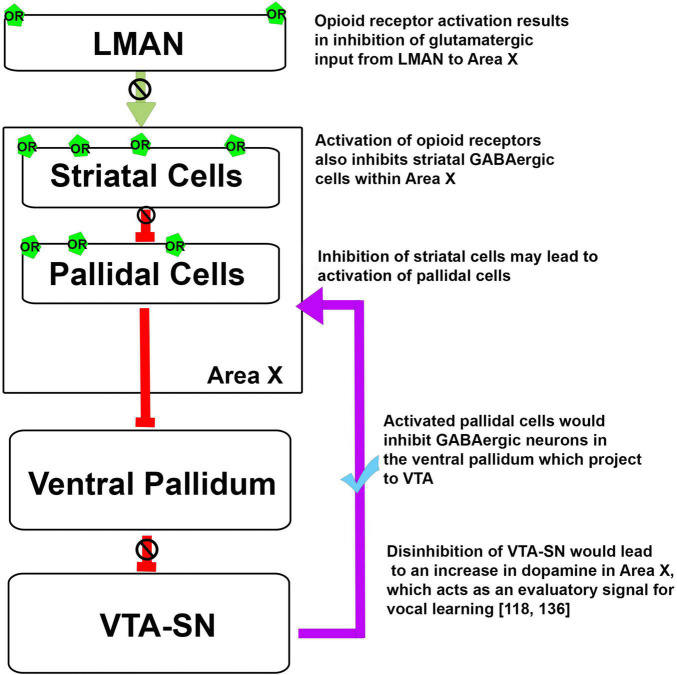
Possible mechanism for the opioid modulation of dopaminergic input to Area X and the regulation of vocal learning. Green arrow, Glutamatergic projection; red arrows, GABAergic projections; violet arrow, dopaminergic projection, OR, opioid receptors.

Earlier studies (Khurshid et al., 2010; Kumar et al., 2019, 2020) have demonstrated that the opioid system can modulate different aspects of singing. Based on the neuroanatomical localization of ORs in the song control areas of oscines, it is possible that these receptors may be involved in vocal learning (Gulledge and Deviche, 1999). As mentioned above, findings from Wada et al. (2006) have demonstrated that there is an increase in the level of the opioid ligand pre-proenkephalin in Area X after singing in juvenile male birds. An increase in the activation of ORs in Area X could potentially lead to a decrease in the activity of MSNs and a disinhibition of pallidal neurons. This would ultimately lead to the disinhibition of the VTA-SN complex and an increase in DA release in Area X, which acts as an evaluatory signal, shown to play an important role in vocal learning (Figure 3; Gadagkar et al., 2016; Xiao et al., 2018). Additionally, ORs are involved in associative learning, modulate dopaminergic signaling, and are differentially expressed in the brain in the developmental phase rather than in the adulthood in mammals and songbirds, which suggests that ORs may influence cognitive processes such as vocal learning.

## Conclusion

A number of studies on different species of mammals and birds have demonstrated that the endogenous opioid system is involved in higher cognitive functions including learning. Whereas the endogenous opioid system has been shown to modulate the motivation to vocalize and also effects the acoustic properties of song in different species of songbirds, recent anatomical findings demonstrate that it is present in components of the AFP. Given that the AFP is involved in learning, is connected to the VTA-SN complex and can influence dopamine release, the endogenous opioid system may potentially modulate vocal learning during the sensitive period. Experiments wherein the ORs are blocked or activated in young songbirds at different time points during the sensitive period would provide further insights into the role of the endogenous opioid system in vocal learning.

## Author Contributions

UAS and SI contributed to scientific discussions and writing leading to this review. SI approved the final manuscript. Both authors contributed to the article and approved the submitted version.

## Conflict of Interest

The authors declare that the research was conducted in the absence of any commercial or financial relationships that could be construed as a potential conflict of interest.

## Publisher’s Note

All claims expressed in this article are solely those of the authors and do not necessarily represent those of their affiliated organizations, or those of the publisher, the editors and the reviewers. Any product that may be evaluated in this article, or claim that may be made by its manufacturer, is not guaranteed or endorsed by the publisher.
